# Adapting Macroecology to Microbiology: Using Occupancy Modeling To Assess Functional Profiles across Metagenomes

**DOI:** 10.1128/mSystems.00790-21

**Published:** 2021-12-07

**Authors:** Angus S. Hilts, Manjot S. Hunjan, Laura A. Hug

**Affiliations:** a Department of Biology, University of Waterloo, Waterloo, Ontario, Canada; Michigan State University

**Keywords:** metagenomics, methane cycling, methanogenesis, methanotrophy, microbial ecology, occupancy modeling

## Abstract

Metagenomic sequencing provides information on the metabolic capacities and taxonomic affiliations for members of a microbial community. When assessing metabolic functions in a community, missing genes in pathways can occur in two ways; the genes may legitimately be missing from the community whose DNA was sequenced, or the genes were missed during shotgun sequencing or failed to assemble, and thus the metabolic capacity of interest is wrongly absent from the sequence data. Here, we borrow and adapt occupancy modeling from macroecology to provide mathematical context to metabolic predictions from metagenomes. We review the five assumptions underlying occupancy modeling through the lens of microbial community sequence data. Using the methane cycle, we apply occupancy modeling to examine the presence and absence of methanogenesis and methanotrophy genes from nearly 10,000 metagenomes spanning global environments. We determine that methanogenesis and methanotrophy are positively correlated across environments, providing a predictive framework for assessing gene absences for these functions. We present this adaptation of macroecology’s occupancy modeling to metagenomics as a tool to quantify the uncertainty in predictions of the presence/absence of traits in environmental microbiological surveys. We further initiate a call for stronger metadata standards to accompany metagenome deposition, to enable robust statistical approaches in the future.

**IMPORTANCE** Metagenomics is maturing rapidly as a field but is hampered by a lack of available statistical tools. A primary area of uncertainty is around missing genes or functions from a metagenomic data set. Here, we borrow an established modeling approach from macroecology and adapt it to metagenomic data sets. Rather than multiple sampling trips to a specific area to detect a species of interest (e.g., identifying a cardinal in a forest), we leverage the enormous amount of information within a metagenome and use multiple gene markers for a function of interest (e.g., subunits of an enzyme complex). We applied our adapted occupancy modeling to a case study examining methane cycling capacity. Our models show methanogens and methanotrophs are both more likely to cooccur than be present in the absence of the other guild. The lack of consistent and complete metadata is a significant hurdle for increasing the statistical rigor of metagenomic analyses.

## INTRODUCTION

Environmental microbiology has been a methods-limited field since its conception. Paradigm shifts in our understanding of microbial diversity and the ecological importance of microbes have come hand in hand with new techniques—from the invention of the microscope to high-throughput sequencing strategies. Each ground-breaking technique then undergoes improvements and refinements and matures into a standard approach. Metagenomics, or total community sequencing (1, 2), has demonstrated that the diversity of life on Earth is far greater than was previously believed (3–6). With metagenomics now a standard method of investigation, and public databases filling with deep-sequencing data sets from sampling locations around the globe, there is a growing need for statistical tests that can be applied to anchor conclusions based on metagenomic data and to understand patterns from incomplete data (see reference 7 for a discussion of current statistical methods).

A current challenge is placing the information obtained from metagenomic sequencing studies into a greater ecological context. Microbial roles in geochemical cycles and their contributions to microbial communities can be predicted from the annotated genes within metagenomes. In generating metabolic predictions for a community, a major challenge is interpreting gene absences. Genes may be absent from a genome or metagenome because they are genuinely not encoded by the genome(s) (i.e., true negatives) or because the sequencing depth or assembly failed to capture genes from the community (i.e., false negatives). There are currently no options for robustly modeling which of these gene absence scenarios is most likely.

This question of how to assess ecological relationships is not new. Macroecologists have studied the relationships of organisms and populations for over a century. The macroecology field has developed a wealth of statistical models, ranging from simple to complex (the founding of the journal *Ecological Modelling* in 1975 is a testament to this focus). In addition, since the 1960s there has been a movement in macroecology to develop and enforce standards for metadata collection and quality assurance (8). As a result, the foundation for ecological modeling is in place, but microbial ecologists must now adapt these ideas to their own data sets.

Our research sought to adapt occupancy modeling to metagenomic data. The occupancy model was developed by Mackenzie et al. (9) to address an important question for any detection-based study; how can missed detections be accounted for? The model addresses the issue that a nondetection could mean that the subject of interest was not present (i.e., a true negative) or that the observer failed to detect it (i.e., a false negative). The underlying idea of occupancy models is that detection can be modeled as two statistical processes, the probability of the species of interest being present at the given site and, given that it is present, the probability that it was observed (9). Two parameters are used to model these, the proportion of sites occupied (denoted Ψ) and the probability of detection given that the species is present at the site (denoted *p*). The value of Ψ can be estimated by counting the number of sites at which the species occurs and dividing it by the total number of sites visited, though in cases of imperfect detection, this will result in an underestimate. The value of *p* can be estimated by revisiting a site at which the species is known to be present multiple times and dividing the number of times the species was observed by the total number of visits. A theoretically perfect model would have unique detection and occupancy parameters for every site, but this model would be extremely challenging to calculate, lack flexibility, and have little, if any, predictive power. Instead, the parameters Ψ and *p* can be generalized (i.e., can be assumed to be equal for all sites and surveys), if the following five assumptions are met:

i) The closure assumption, which states that there is no chance of the occupancy state changing between sampling occasions for the site, within the same season.
ii) The probability for occupancy is the same across all sites or is otherwise modelled with appropriate covariates.
iii) The probability for detection is the same across all sites or is otherwise modelled with appropriate covariates.
iv) The detection at each site is independent of detection at other sites.
v) There are no false positives.

These assumptions must be systematically considered when reinterpreting the model for sequence-based data sets.

The original occupancy model was designed to assess the occupancy of a single species, but its applications have since been expanded. Occupancy models that accommodate false positives have been developed (10, 11). Multispecies models allow exploration of other species of interest (12), with more recent models allowing multispecies modeling without assuming a dominant partner (13).

Here, we apply occupancy models to metagenomic data sets. We develop a method for deriving replicate sampling for functions of interest from a single metagenome and assess each of the five model assumptions under metagenomic data. We applied this approach as a proof-of-principle to the global methane cycle, using a data set of 9,629 metagenomes spanning global environments to assess the proportion of environments that contain the capacity for methanogenesis and methanotrophy. From these metagenomes, we identified and curated markers for methanogenesis (McrABG) and methanotrophy (PmoABC) as proxies for predicting these functions in an environment, and assessed cooccurrence patterns for these two critical components of the methane cycle.

## RESULTS AND DISCUSSION

### Occupancy modeling as applied to metagenomes.

In adapting occupancy modeling to metagenomics, the question for microbial ecologists becomes one of identifying parallels between macroecological and microbiological data sets or systems. There are several key aspects that need to be considered here, and in each of these aspects, the solutions may not be universal or applicable to all studies. First, the definition of a site needs to be considered. In addition to this, how a sample unit is defined is important, particularly when considering the cost and labor involved in resampling a metagenome. Finally, the applicability of the assumptions underlying occupancy modeling must be determined.

**Site definition.** There are multiple possible definitions of a site, with the simplest being defining a single sample taken for metagenomics as a site. This has the advantage of unambiguously delineating the site but also poses some disadvantages. For example, a hypothesis exploring cooccurrence of two or more species may not require that the species be present in exactly the same sample but that they be present within the same system (e.g., a soil core, lake depth profile, or host gastrointestinal system). In these cases, it may be better to define a geographic location or geographic feature as a single site (e.g., the same forest, lake, host), aggregating all associated metagenomes for the defined site. Ultimately, the definition of a site will be dependent on the specific questions of each study, but a clear definition is an important preanalysis requirement.

**FIG 1:**
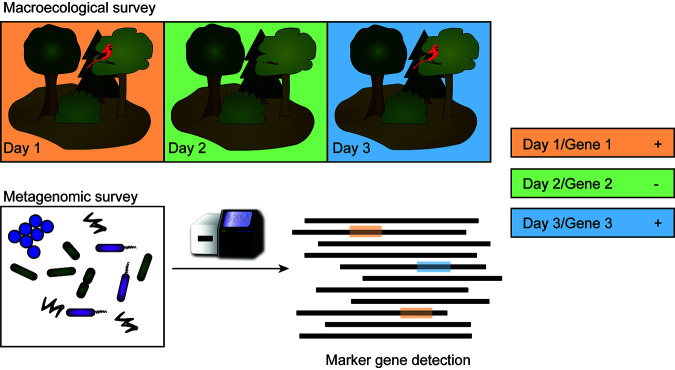
Schematic depicting connections between occupancy sampling for macroecologists compared to the proposed method for metagenomic data sets. Where macroecological occupancy sampling requires repeated observation (days 1, 2, 3) of the same environment (forest) for the presence/absence of a species of interest (bird), we propose using molecular markers (genes 1, 2, 3) for an activity or lineage of interest that allow multiple observations of the presence/absence from within a single metagenomic data set.

**Sample unit.** Another key component of occupancy modeling is that resampling a site yields an estimation of the observer’s ability to detect the subject of interest (the variable *p* within the models). Thus, a definition of a resampling event, or replicate of a surveyed, site must be established. When working with metagenomes, there are several issues with resampling. It is expensive, labor-intensive, and time-consuming to obtain samples, isolate DNA, and perform sequencing analyses. Furthermore, it is not always clear what might constitute a replicate sample. Microbial communities mere centimeters apart can be substantially and meaningfully different (14), and by sampling a site, community composition can be changed due to disturbances. In contrast to macroecological observational surveys, however, each metagenome provides an enormous volume of data (Fig. 1), containing information from the genomic sequences of hundreds or thousands of microbial populations (15, 16). Given the depth of information available, we instead propose resampling a single metagenome for multiple genetic markers of a function or group of interest. Each independent marker is then considered a replicate survey for the target function or taxonomic group.

From this, a critical step in the application of occupancy models to microbial ecology becomes the decision of which marker genes to use. Genes must be universally and uniquely associated with the target function or taxonomic group. For a target function, an obvious choice is genes encoding different, required subunits of an enzyme complex associated with the function of interest. For a taxonomic group, core genes specifically and universally associated with the group of interest would be required. Occupancy models are explicitly designed to avoid the problem of false negatives, but as a result they are biased toward false positives. There are adaptations to occupancy modeling that handle false positives more robustly (10, 11), but false positives within sequencing data are potentially more likely than for macroecological surveys (e.g., confounding of closely related protein families), so whether a marker can be separated into true or false positives should be a first consideration. How false positives are filtered out will depend on the selected marker genes, but considerations such as active site, conserved residues, secondary and tertiary structure modeling, length requirements, homology to characterized proteins, and phylogenetic placement can be applied. Ultimately, the decision of which markers can be used, and what curation is required, will be study-specific. In our case study below, the methyl-coenzyme M reductase (MCR) complex is used as a marker for methanogenesis, despite its activity in anaerobic oxidation of methane in the ANaerobic MEthanotrophic archaea (ANME) organisms; our curation thus required us to remove any MCR detected that were associated with ANME to ensure we were targeting the correct function of interest. The use of multiple marker genes as “resamplings” provides the advantage that multiple metagenomic samples are not needed and also means that several of the assumptions of occupancy models are met “for free” (see next).

**Occupancy modeling assumptions.** Occupancy modeling separates successful detections into two processes; first, the species of interest must be present at the survey site, and second, given that it is present, it must be successfully detected. These processes are modeled as two probabilities, denoted Ψ and *p* (9). The first parameter, Ψ, represents the proportion of occupied sites, while the latter, *p*, represents the probability that, given the species is present, it is successfully detected. A theoretically perfect model could have a unique detection and occupancy parameter for each site, but this would sacrifice flexibility. Instead, the model can be generalized using certain assumptions, which restrict these parameters to single values across all sites. Mackenzie et al. (9) proposed five assumptions that, if met, allow for this generalization to be applied. In brief, each assumption seems reasonable to apply to metagenomic data sets, but there are specific points that require consideration or definition.

**(i) The closure assumption, which states that there is no chance of the occupancy state changing between sampling occasions for the site, within the same season.** The first assumption is met without ambiguity when using multiple genetic markers from a single metagenome as the sampling units. Detection of these markers is conducted on the same metagenome, from the same extracted DNA, taken at the exact same time. This is an advantage of metagenomic data sets compared to macroecological data sets, where the observer must return to a site over multiple separate occasions.

**(ii) The probability for occupancy is the same across all sites, or is otherwise modeled with appropriate covariates and (iii) the probability for detection is the same across all sites or is otherwise modeled with appropriate covariates.** The second (ii) and third (iii) assumptions are more challenging. It is unlikely that detection and occupancy are uniform across all sites. In particular, the occupancy state is expected to change as a result of different environmental conditions, such as pH, temperature, and organic carbon content of the site. It may be more reasonable to assume that detection probabilities are uniform, but even this is unlikely. Factors affecting detection probabilities include challenges in DNA extraction for difficult matrices or resistant microorganisms as well as variations due to changing sequencing technologies, where greater sequencing depths enable better assemblies and a higher likelihood of detecting a function or taxon at low abundance. It is possible to separately estimate detection probability for each of these factors (DNA extraction, sequencing depth) to assess whether different sampling techniques may be more or less capable of detecting the function of interest. Alternatively, variation in detection and occupancy can be modeled with covariates. In this context, a covariate might be metagenome size, pH, total organic carbon, elevation above sea level, or other factors that would influence occupancy and detection for the trait or taxon of interest. Identifying useful covariates was one of the major challenges in our methane cycle case study, described below. Metadata reported for metagenomes was sporadic and incomplete, meaning that there were very few variables that could be used as covariates. Better data deposition standards have the potential to rapidly remedy this problem.

**(iv) The detection at each site is independent of detection at other sites.** Assumption iv is a reasonable assumption for metagenomic data. Unless samples were cross-contaminated, metagenomes should have no impact on the content of other metagenomes and thus can be treated as independent from one another. This holds true if aggregating metagenomes into larger-scale samples (e.g., a soil core), as long as each set of metagenomes is from samples/sites that do not meaningfully interact.

**(v) There are no false positives.** This assumption also poses a challenge. The best solution is to employ procedures that carefully curate the detections prior to modeling, as described above. Searching against established databases, the use of annotation pipelines, multiple sequence alignments, and phylogenetics can be used to minimize the number of false positives. Further case-specific information can be valuable, such as identifying key conserved residues at active sites or motifs thought to be conserved across all members of the group of interest. By incorporating this sort of information and applying rigorous filtering to the data, false positives can be minimized or eliminated.

### Occupancy modeling and the global methane cycle.

We took our theorized definitions of sites, sampling units, and the assumptions required for occupancy modeling and applied them to a case study examining the cooccurrence of methanogenesis and methanotrophy across global environments.

Two microbial groups are the main controls on biological methane cycling and the flux of methane emissions. The majority of methane emissions originate from methanogenic archaea (17). The other group of organisms implicated in the methane cycle are the methane oxidizers. Methane-oxidizing organisms largely fall into two categories: the bacterial methanotrophs (18) and the archaeal anaerobic oxidizers of methane (AOM; 19, 20). Understanding the cooccurrence patterns of methanogens and methanotrophs across global environments would provide insight into methane emission fluxes and spotlight regions where microbial control of methane emissions is tipped toward higher emissions (i.e., sites with methanogens and no associated methanotrophs). Using 9,629 publicly available metagenomes from a wide variety of global environments, we applied occupancy modeling to metagenomic data to assess the cooccurrence of methanogenesis and methanotrophy in the context of geographic location and environment type.

In defining a site for the occupancy models, we tested three approaches. First, we tested the naive approach of each metagenome equating to a single site. However, methanogens and methanotrophs differ in their oxygen requirements; methanogens are obligately anaerobic, whereas methanotrophs are generally understood to be microaerophilic (18, 21), though this paradigm is shifting with increased reports of methanotrophy in anoxic conditions (22, 23). Given this difference, we would not generally expect methanogens and methanotrophs to occur at the exact same location, but their presence within the same system would be informative. To address this, our second approach was to aggregate metagenomes from the same geographic coordinates (latitude and longitude) as a single site, meaning soil cores and depth profiles were merged. Our third and final approach was to separate the aggregated metagenomes in the second approach if their environmental coding differed between environmental, engineered, and host-associated.

For sampling units, we required a set of genes that could act as proxies for function and be used to emulate resampling an environment, within a single metagenome. We selected subunits of the catalytic enzyme complexes responsible for the final step of methanogenesis and the first step of methane oxidation—methyl-coenzyme M reductase (MCR; subunits McrABG) and particulate methane monooxygenase (pMMO; subunits PmoABC). Both enzyme complexes are built from three distinct and necessary subunits. All known methanogens contain *mcr* genes in their genomes (24, 25), which encode the MCR complex responsible for the final step in methane formation (26, 27). The *mcr* operon contains a suite of genes, but the three that encode the MCR complex are *mcrA*, *mcrB*, and *mcrG*, which encode the α, β, and γ subunits, respectively (28). The MCR enzyme complex is an α_2_β_2_γ_2_ hexamer (29). The *mcrABG* genes are frequently used in combination as methanogenic markers (30–32) and can be used in place of 16S rRNA genes to infer phylogeny of methanogens (33). The oxidation of methane is catalyzed by methane monooxygenases (MMO), which have two forms, soluble and particulate (34). The soluble form (sMMO) is localized to the cytoplasm, while the particulate form (pMMO) is membrane bound (35). We chose the pMMO enzyme over sMMO as a methanotrophic marker because the soluble form usually occurs alongside the particulate form, but the reverse is not necessarily true. The pMMO complex is encoded by the *pmoCAB* operon (36, 37). These genes, especially *pmoA*, are used as functional markers for aerobic methanotrophy (35, 38, 39). While the *pmo* operon is present in most methanotrophs, it has recently been found to be absent in some species (37, 38; for a review see reference 39), meaning this marker may lead to false negatives. Another source of false negatives in our analyses is anaerobic oxidation of methane by the ANME archaea (19, 20). The ANME archaea use the methanogenic pathway in reverse, including McrABG, to catalyze the oxidation of methane (40–42). These methane-oxidizing archaea are fascinating and likely important players in methane cycling (43), but there is growing evidence the ANME can also produce methane (44), meaning their McrABG are ambiguous markers for methane cycling, and we wanted a straightforward initial case study. As a result, for the work described below, our focus was on oxidation of methane performed by bacteria. The main relevance of the ANME archaea for this research was that any McrABG proteins that were closely associated with the ANME MCR were removed from the methanogenesis data sets. Excluding sMMO and ANME MCR from our methanotrophic survey means we have not captured the total methane oxidation capacity from the surveyed environments, but for this proof-of-principle exercise, the advantages of having an equal number of marker genes and a single set of markers per function of interest outweighed the potential for false negatives.

Reviewing the 5 assumptions to allow for generalized Ψ and *p* parameters in our occupancy model, assumption i is met, as each metagenome or aggregated set of metagenomes was static in time and searched simultaneously for the six marker genes. Assumptions ii and iii are not met with the raw data, and so we used covariates to model factors impacting occupancy (ii) and detection (iii) probabilities. Assumption iv is met, as detections between sites are independent, given the reasonable assumption that each metagenome stemmed from independent DNA extractions from isolated samples (e.g., sample handling minimized cross-contamination). Assumption v, that there are no false positives, required significant data set curation of our candidate marker genes.

**FIG 2:**
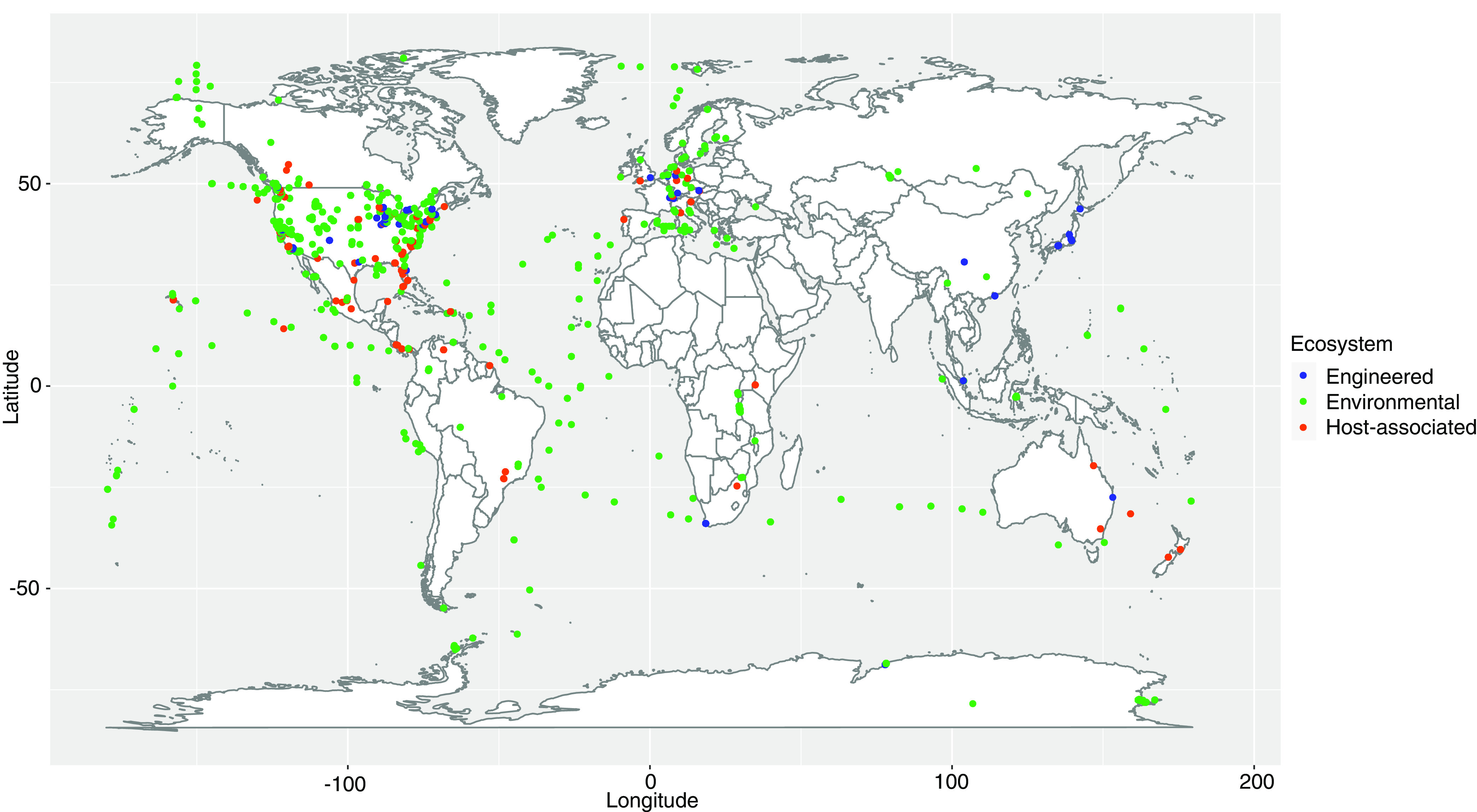
Geocoordinates associated with all metagenomes examined for methanogenesis and methanotrophy marker enzymes. All continents were included within this survey, but there is a clear sampling bias toward North America and Europe. The map was generated using the R package “maps,” which is open source and in the public domain.

The data set used consisted of 9,629 metagenomes from across every continent on Earth (Fig. 2). The metagenomes were classified into three broad categories; 80.6% were from environmental samples, 13.3% were host-associated samples, and 6.2% were from engineered environments. From these data sets 261,869 sequences were identified as candidate matches to the target genes (based on KEGG KO annotations). After size-filtering sequences to remove partial and truncated sequences, 59,390 candidate sequences remained. Filtering using DIAMOND BLASTp further reduced the set, leaving 29,308 sequences. Finally, after manual assessment for phylogenetic congruence, 27,730 of the initial sequences remained. This curation was important to minimize false positives to satisfy assumption v. Following curation, the frequencies of each complex’s subunits were similar within different environmental categories (Fig. S1 and S2).

10.1128/mSystems.00790-21.1 FIG S1 Proportions (as percentage) of environment type containing *mcr* genes. Numbers in parentheses indicate the number of metagenomes included in the given category. Download FIG S1, PDF file, 0.1 MB. Copyright © 2021 Hilts et al. 2021 Hilts et al. https://creativecommons.org/licenses/by/4.0/ This content is distributed under the terms of the Creative Commons Attribution 4.0 International license.

10.1128/mSystems.00790-21.2 FIG S2 Proportions (as percentage) of environment type containing *pmo* genes. Numbers in parentheses indicate the number of metagenomes included in the given category. Download FIG S2, PDF file, 0.1 MB. Copyright © 2021 Hilts et al. 2021 Hilts et al. https://creativecommons.org/licenses/by/4.0/ This content is distributed under the terms of the Creative Commons Attribution 4.0 International license.

Single species models were run for each of the two functions (methanogenesis and methanotrophy) (Table 1). In nearly all cases, the null model (i.e., models run without any covariates) explained the least variability in the data. The available covariates were ecosystem type, latitude, and date of metagenome deposition. Where applicable, ecosystem type as a covariate for the occupancy state contributed to the best model (Table 1). In addition to this, the latitude seemed to offer some small improvement. The square-root-transformed add date, counted as days since 1 January 2006, improved models when used as a covariate for the detection probability (Table 1).

**TABLE 1:** Occupancy model parameters for MCR and pMMO data sets^*a*^

Model		MCR		pMMO	
	No. of parameters	AIC	ΔAIC	AIC	ΔAIC
Metagenomes as sites (*n* = 9,420)					
*p* ∼ 1, Ψ ∼ ecosystem	4	9,691.23	0.00	15,669.51	0.00
*p* ∼ sqrt(numeric.add.date), Ψ ∼ latitude	4	9,936.71	245.47	15,705.84	36.33
*p* ∼ 1, Ψ ∼ latitude	3	9,958.31	267.08	15,929.51	260.00
*p* ∼ sqrt(numeric.add.date), Ψ ∼ 1	3	9,987.42	296.18	15,830.36	160.85
*p* ∼ 1, Ψ ∼ 1	2	10,007.15	315.91	16,053.68	384.17
Aggregated by geocoordinates and environment (1,229 sites)					
*p* ∼ 1, Ψ ∼ ecosystem	4	1,555.15	0.00	2,284.47	0.00
*p* ∼ 1, Ψ ∼ latitude	3	1,594.58	39.43	2,329.47	45.00
*p* ∼ sqrt(numeric.add.date), Ψ ∼ Latitude	4	1,596.74	41.59	2,308.65	24.18
*p* ∼ 1, Ψ ∼ 1	2	1,619.65	64.50	2,330.83	46.36
*p* ∼ sqrt(numeric.add.date), Ψ ∼ 1	3	1,621.81	66.66	2,310.07	25.60
Aggregated by geocoordinates only (1,202 sites)					
*p* ∼ 1, Ψ ∼ latitude	3	1,578.66	0.00	2,297.88	0.00
*p* ∼ 1, Ψ ∼ 1	2	1,605.03	26.37	2,299.55	1.67

a Separate models were developed for each aggregated set of global metagenomes under each of the possible Ψ values, with *p* ∼ 1 (intercept only model) or as the square root (sqrt) of the metagenome’s add date (as a count from 1 January 2006). Results are ordered by ascending AIC for MCR. AIC, Akaike information criterion.

**FIG 3:**
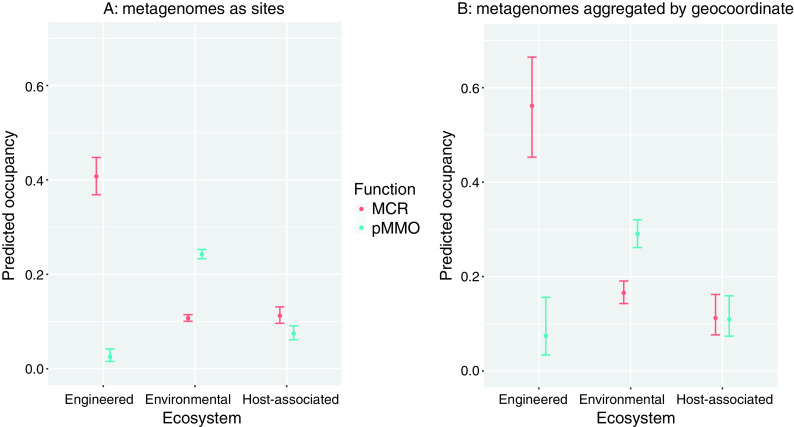
Predicted proportion of sites occupied for both MCR and pMMO. (A) The unaggregated metagenomes; (B) aggregated metagenomes where the geocoordinate and ecosystem type were the same. The model used for both plots was *p ∼* 1, Ψ ∼ ecosystem. Error bars indicate 95% confidence intervals for the estimated occupancies.

In general, the models predicted that methanogens occupied a larger portion of engineered sites than methanotrophs. In contrast, a higher proportion of environmental sites were predicted to be occupied by methanotrophs than methanogens, and the proportion of host-associated sites occupied by each group was similar (Fig. 3). For both functional groups, the proportion of sites occupied appeared to increase with latitude. Interestingly, this trend followed from extreme southern latitudes through to the extreme northern latitudes, rather than increasing from the equator toward both poles (Fig. S3). This trend may reflect a strong geographic bias in sampling. The majority of available metagenomes were from samples taken from locations in North America and western Europe (Fig. 2), which cover a similar range of latitudes. In practice, this may impact the model estimates of occupancy, biasing them toward detection in better-covered regions of the map. Finally, the estimated detection probability increased with the square-root-transformed dates (Fig. S4). This may not mean there was an increase in prevalence of these metabolisms over time but, rather, that this covariate captures underlying differences in the data that would be better or more precisely captured by other covariates that were not accessible to us due to incomplete metadata for the submitted metagenomes. For example, the observed increase may be tied to advances in technology and/or be due to increasing metagenome sizes over time leading to higher detection probabilities of lower-abundance functions.

10.1128/mSystems.00790-21.3 FIG S3 (A to C) Predicted proportion of sites occupied versus latitude when using metagenomes as individual sites (A), metagenomes aggregated by geocoordinate and ecosystem type as sites (B), and metagenomes aggregated by geocoordinates only (C). Shaded regions represent 95% confidence intervals for the predicted occupancy (solid lines). Note the smaller confidence intervals in panel A, likely due to inflated sampling, since a given geocoordinate may have multiple samples, which is not the case for panels B and C. Download FIG S3, PDF file, 1.3 MB. Copyright © 2021 Hilts et al. 2021 Hilts et al. https://creativecommons.org/licenses/by/4.0/ This content is distributed under the terms of the Creative Commons Attribution 4.0 International license.

10.1128/mSystems.00790-21.4 FIG S4 Detection probability (*p*) versus date for unaggregated data, as predicted by occupancy models. Dates were converted to a numeric format (counted from 1 January 2006) and square-root-transformed; these were used as the only covariate for prediction. Shaded regions indicate 95% confidence intervals for the estimated detection probabilities (solid lines). Download FIG S4, PDF file, 0.3 MB. Copyright © 2021 Hilts et al. 2021 Hilts et al. https://creativecommons.org/licenses/by/4.0/ This content is distributed under the terms of the Creative Commons Attribution 4.0 International license.

**FIG 4:**
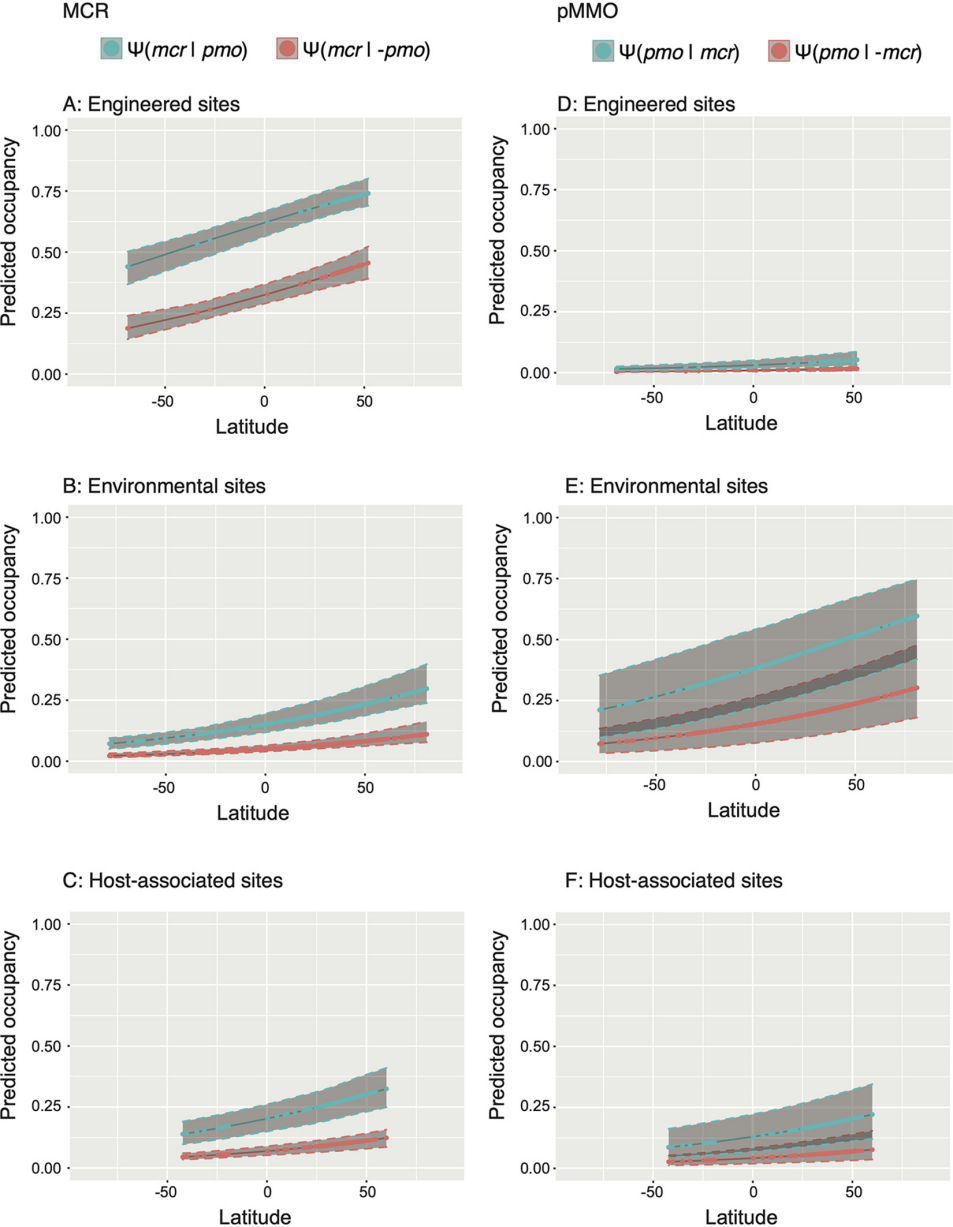
Predicted occupancy for functions of interest given the presence (blue) or absence (red) of the other function. Shaded regions represent 95% confidence intervals for the estimates (solid lines). All sites are unaggregated metagenomes. The model used was Ψ[mcr] ∼ ecosystem, Ψ[pmo] ∼ ecosystem + sqrt(numeric.add.date), Ψ[mcr:pmo] ∼ ecosystem. (A to C) The estimated occupancy for MCR; (D to F) show that of pMMO.

In the case of the multispecies models, the ecosystem covariate was found to have the strongest influence in explaining the data, regardless of the data set used (Table 2). In all cases, the presence of the other functional group in the model increased the estimated proportion of sites occupied for the other group (Fig. 4, Fig. S5). For the unaggregated data, the occupancy in MCR increased if pMMO was present for the entire range of latitudes, in all three environment types (Fig. 4). According to the models, this prediction was most robust for engineered sites. The confidence intervals for both environmental and host-associated sites were much broader, but the same trends were observed. The same trend was observed for pMMO, which increased in occupancy if MCR was present. However, the occupancy of pMMO in engineered sites was very low regardless of the occupancy of MCR, possibly due to a bias toward anaerobic systems for the engineered environments. More than a third (36%) of engineered metagenomes were labeled as deriving from “anaerobic bioreactors,” where the system is manipulated to maintain anoxic conditions which would exclude the aerobic methanotrophs. The aggregated data sets were similar in pattern compared to the unaggregated data. However, the confidence intervals were much larger, most notably for the occupancy of pMMO at environmental sites (Fig. S5). The data that were aggregated solely by geocoordinates showed the clearest trend where the occupancy of each function increased when the other function was present (Fig. S6). A hypothesis of this work was that methanotrophs would be more likely to occur at sites containing methanogens or would occur at close proximity to sites containing methanogens. The opposite case, where a methanogen would be more likely to occur if a methanotroph was present, is not anchored in our understanding of the biology of these organisms. This makes our model results interesting, since both scenarios are predicted to be true. It may be that external variables controlling the presence or absence of each group are shared, and so the two functional groups coincide because of limitations to their distributions. The size of the confidence intervals across all trials prevents strong conclusions from being drawn. We note that sample date was not considered when aggregating data sets; of the 1,093 aggregated data sets based on geographical coordinates, 285 (26%) include samples from different dates. Refining this aggregation might improve confidence intervals on these occupancy estimates.

**TABLE 2:** Multispecies occupancy models for MCR and pMMO across global metagenomes^*a*^

Model	No. of parameters	AIC	ΔAIC
Metagenomes as sites (*n* = 9,420 sites)			
Ψ[mcr] ∼ ecosystem, Ψ[pmo] ∼ ecosystem + sqrt(numeric.add.date), Ψ[mcr:pmo] ∼ ecosystem	12	24,803.63	0
Ψ[mcr] ∼ ecosystem + latitude, Ψ[pmo] ∼ ecosystem + latitude, Ψ[mcr:pmo] ∼ 1	11	24,919.92	116.28
Ψ[mcr] ∼ ecosystem, Ψ[pmo] ∼ ecosystem, Ψ[mcr:|pmo] ∼ ecosystem Ψ[mcr] ∼ ecosystem, Ψ[pmo] ∼ ecosystem, Ψ[mcr:pmo] ∼ 1	11	25,043.00	239.37
Ψ[mcr] ∼ sqrt(numeric.add.date), Ψ[pmo] ∼ sqrt(numeric.add.date), Ψ[mcr:pmo] ∼ sqrt(numeric.add.date)	9	25,076.89	273.26
Ψ[mcr] ∼ latitude, Ψ[pmo] ∼ latitude, Ψ[mcr:pmo] ∼ latitude Ψ[mcr] ∼ 1, Ψ[pmo] ∼ 1, Ψ[mcr:pmo] ∼ ecosystem	8	25,622.46	818.83
Ψ[mcr] ∼ 1, Ψ[pmo] ∼ 1, Ψ[mcr:pmo] ∼ 1	8	25,728.01	924.38
Ψ[mcr] ∼ ecosystem+ sqrt(numeric.add.date),	7	25,792.37	988.73
Ψ[pmo] ∼ ecosystem+ sqrt(numeric.add.date), Ψ[mcr:pmo] ∼ 1	5	25,882.22	1,078.59
Ψ[mcr] ∼ 1, Ψ[pmo] ∼ 1	11	35,276.17	10,472.54
Ψ[mcr:pmo] ∼ ecosystem	13	35,280.17	10,476.54
Aggregated by geocoordinates and environment (1,229 sites)			
Ψ[mcr] ∼ ecosystem, Ψ[pmo] ∼ ecosystem + sqrt(numeric.add.date), Ψ[mcr:pmo] ∼ ecosystem	12	3,745.72	0
Ψ[mcr] ∼ ecosystem + latitude, Ψ[pmo] ∼ ecosystem + latitude, Ψ[mcr:pmo] ∼ 1	11	3,757.98	12.26
Ψ[mcr] ∼ ecosystem, Ψ[pmo] ∼ ecosystem, Ψ[mcr:pmo] ∼ ecosystem Ψ[mcr] ∼ ecosystem, Ψ[pmo] ∼ ecosystem, Ψ[mcr:pmo] ∼ 1	11	3,776.41	30.69
Ψ[mcr] ∼ sqrt(numeric.add.date), Ψ[pmo] ∼ sqrt(numeric.add.date), Ψ[mcr:pmo] ∼ sqrt(numeric.add.date)	9	3,781.61	35.89
Ψ[mcr] ∼ latitude, Ψ[pmo] ∼ latitude, Ψ[mcr:pmo] ∼ latitude Ψ[mcr] ∼ 1, Ψ[pmo] ∼ 1, Ψ[mcr:pmo] ∼ ecosystem	8	3,878.95	133.23
Ψ[mcr] ∼ 1, Ψ[pmo] ∼ 1, Ψ[mcr:pmo] ∼ 1	8	3,885.95	140.23
Ψ[mcr] ∼ ecosystem+ sqrt(numeric.add.date),	7	3,888.03	142.32
Ψ[pmo] ∼ ecosystem+ sqrt(numeric.add.date), Ψ[mcr:pmo] ∼ 1	5	3,908.36	162.64
Ψ[mcr] ∼ 1, Ψ[pmo] ∼ 1	11	5,131.21	1,385.49
Ψ[mcr:pmo] ∼ ecosystem	13	5,135.21	1,389.49
Aggregated by geocoordinates only (1,202 sites)			
Ψ[mcr] ∼ latitude Ψ[pmo] ∼ latitude, Ψ[mcr:pmo] ∼ 1	7	3,839.91	0
Ψ[mcr] ∼ latitude, Ψ[pmo] ∼ latitude, Ψ[mcr:|pmo] ∼ latitude	8	3,841.47	1.56
Ψ[mcr] ∼1, Ψ[pmo] ∼ 1, Ψ[mcr:pmo] ∼ latitude	6	3,851.49	11.58
Ψ[mcr] ∼ 1, Ψ[pmo] ∼ 1, Ψ[mcr:pmo] ∼ 1	5	3,865.21	25.39

a Separate models were developed for each aggregated set of global metagenomes under each of the possible Ψ values, with *p* ∼ 1 (intercept-only model for species-specific detection probabilities). AIC, Akaike information criterion.

10.1128/mSystems.00790-21.5 FIG S5 Predicted occupancy for functions of interest given the presence (blue) or absence (red) of the other function. Shaded regions represent 95% confidence intervals for the estimates (solid lines). All sites are metagenomes aggregated by their respective geocoordinates and ecosystem types. (A to C) Estimated occupancy for MCR; (D to F) that of pMMO. Download FIG S5, PDF file, 1.3 MB. Copyright © 2021 Hilts et al. 2021 Hilts et al. https://creativecommons.org/licenses/by/4.0/ This content is distributed under the terms of the Creative Commons Attribution 4.0 International license.

10.1128/mSystems.00790-21.6 FIG S6 Predicted occupancy for functions of interest given the presence (blue) or absence (red) of the other function, with all sites as metagenomes aggregated by their respective geocoordinates and with latitude as a covariate. Shaded regions represent 95% confidence intervals for the estimates (solid lines). Download FIG S6, PDF file, 1.2 MB. Copyright © 2021 Hilts et al. 2021 Hilts et al. https://creativecommons.org/licenses/by/4.0/ This content is distributed under the terms of the Creative Commons Attribution 4.0 International license.

This initial application of occupancy modeling has generated a series of interesting hypotheses around the interconnection of methanogens and methanotrophs within the global methane cycle. The positive occupancy relationship of MCR in the presence of PMO warrants further study, as does the connection of occupancy to latitude. Application of this adaptation of occupancy modeling to other global geochemical cycles is likely to identify similarly interesting trends and generate hypotheses to guide future sampling, sequencing, and analysis efforts.

What this proof-of-principle study also demonstrates is an ability to associate confidence intervals with metagenomic predictions and to temper conclusions based on statistical analyses. Given a new metagenome, the best-fitting model can be applied to predict the presence/absence of one of the traits of interest, given the presence/absence of the other(s) and values of the associated covariates. If a trait is not identified in the metagenome, the model prediction provides a quantified likelihood as to whether this is a true or false negative. For researchers applying occupancy modeling to smaller-scale data sets (e.g., trait occupancy and cooccurrence across a connected system such as a municipal landfill or aquifer), the models will provide quantification of uncertainty around trait presence/absence, likely with narrower confidence intervals than were seen in this global survey. If applied with care in selecting covariates and definitions of site and sample, this method provides a way to robustly model whether a true or false negative is the more likely scenario when a trait is not annotated within a metagenome. Occupancy modeling enables hypotheses on microbial metabolism to be anchored in statistical analysis and is a valuable addition to the growing toolkit for metagenome statistics. This move to greater rigor in hypothesis generation is a necessary and important addition to the maturation of metagenomics as a field.

### Metagenomic metadata is a limiting factor for statistical analyses.

While our models predicted interesting cooccurrence patterns, we were constrained by the lack of metadata available for the metagenomes of interest. The accuracy of occupancy modeling is specifically strengthened by continuous covariates that can be used to assess how detectability and occupancy vary as some external parameter does (9). The metadata associated with the metagenomic data sets used here included very few numerical covariates, which were often incomplete. Our models showed occupancy trends associated with the available numerical covariates, though we think some of the observed trends may be driven by sampling biases, particularly for the latitude covariate. Having more covariates would help to deconflate the underlying confounding factors that are driving these biases or allow selection of covariates with lower bias levels. Metagenomic data sets with curated metadata are good candidates for occupancy modeling. An advantage to microbial surveys over macroecological ones is that different sites can be located very close to each other (e.g., soil core depth samples), for which detailed metadata can be collected, including variables that are expected to explain changes in the microbial community (e.g., pH, electrical conductivity, dissolved organic carbon). Our case study was purposefully ambitious; a global-scale examination is not a requirement for the application of occupancy modeling to assess the presence, absence, and cooccurrence of functions.

To further strengthen statistical assessment of metagenome data, it would be useful to implement better metadata deposition standards. This would not require new or standardized sampling protocols, which would be near impossible to implement across environments. Instead, database administrators could more strongly encourage deposition of more metadata, to be uploaded alongside sequence data sets. A way forward might be requiring a suite of basic metadata for data deposition (e.g., pH, temperature, oxygen content) as well as enforcing the use of a hierarchical classification system for categorizing environments (e.g., the ENVironment Ontology system [ENVO]). A process for requesting exceptions based on resource availability would be required, to ensure metadata reporting does not represent a new barrier to less well-resourced laboratories, potentially exacerbating the observed geographic bias. If the default of providing metadata is faster and simpler than requesting an exception, uptake might improve significantly. Applying a standardized environment classification system for all deposited metagenomic data would not increase the cost or complexity of sampling, and would be accessible for all research groups regardless of the resources available, making this a means to substantially improve metadata at a minimum burden.

### Conclusions.

Perfect detection is unlikely to ever be fully achieved in field ecology. The need to properly account for false negatives in detection surveys motivated the development of the occupancy model by Mackenzie et al. in 2002 (9). The method outlined here provides a novel approach for microbial ecologists to apply macroecological occupancy models to microbial metagenomic data sets and to assess microbial interactions on a global scale. The advantage to this approach is that it enables researchers to study a hypothesis of interest using statistical replicates, without the need for expensive resampling. We successfully applied occupancy modeling to metagenomic data sets, generating predictions about the cooccurrence patterns of methanogenesis and methanotrophy across global environments. None of the covariates available with these data were particularly compelling, and yet each improved either the occupancy or detection probability model fit when applied. Occupancy modeling on metagenomics provides a statistical framework for assessing the cooccurrence of key functions in global geochemical cycles and provides a method to assess the likelihood of a missing annotation being a true or a false negative. For metagenomic research to continue to evolve statistical rigor, better data-deposition standards are required to ensure that relevant metadata are available to researchers.

## MATERIALS AND METHODS

### Reference protein retrieval.

Reference sequences for the proteins of interest (methanogenesis: McrA, McrB, McrG; methanotrophy: PmoA, PmoB, PmoC) as well as known homologs with alternative functions (AmoA, AmoB, AmoC) were retrieved from the Genome Taxonomy Database (GTDB; 45) using AnnoTree (46; accessed 11 February 2019) and the Kyoto Encyclopedia of Genes and Genomes Orthology (KO) based on KO numbers (Table 3; 53). Data were downloaded in CSV format. Any sequences that were metagenome-derived or for which taxonomy was not resolved to the species level were removed, unless there was supporting literature for the protein as a true representative of the function of interest. Sequences were imported into Geneious v. 11.0.2 (54), aligned with MUSCLE v. 3.8.425 (47), and maximum likelihood trees were inferred using FastTree v. 2.1.5 (48) to assess the quality of the reference sets. The final reference data sets’ accession numbers and host names are listed in Table S1.

**TABLE 3:** Protein sequences identified for reference sets (all) as well as from annotated metagenomes (MCR and pMMO complexes only)^*a*^

Protein symbol	Gene name	KO identifier
McrA*	Methyl-coenzyme M reductase alpha subunit	K00399
McrB	Methyl-coenzyme M reductase beta subunit	K00401
McrG	Methyl-coenzyme M reductase gamma subunit	K00402
PmoA	Particulate methane/ammonia monooxygenase subunit A	K10944
PmoB*	particulate methane/ammonia monooxygenase subunit B	K10945
PmoC	Particulate methane/ammonia monooxygenase subunit C	K10946
AmoA	Ammonia monooxygenase subunit A	K10944
AmoB*	Ammonia monooxygenase subunit B	K10945
AmoC	Ammonia monooxygenase subunit C	K10946

a Note that Pmo and Amo proteins share KO identifiers. Asterisks signify the active site-containing subunit of the complexes.

10.1128/mSystems.00790-21.7 TABLE S1 Organism names and accession numbers for the reference sets of *mcrABG* and *pmoABC*. Download Table S1, CSV file, 0.1 MB. Copyright © 2021 Hilts et al. 2021 Hilts et al. https://creativecommons.org/licenses/by/4.0/ This content is distributed under the terms of the Creative Commons Attribution 4.0 International license.

### Metagenome-derived gene collection and curation.

Data were retrieved from the Joint Genome Institute’s portal for Integrated Microbial Genomes and Microbiomes (JGI IMG/M; accessed between 14 December 2018 and 22 February 2019). All sequences annotated with KOs of interest (Table 3, top) were downloaded in FASTA amino acid format along with metadata tables for the metagenomes from which they were derived. Sequences retrieved from IMG/M were first filtered based on size in a protein-specific manner, with passing sequences falling between a minimum length of the smallest sequence in the reference set less 50 amino acids and a maximum length of 50 amino acids longer than the longest reference sequence for that protein. Sequences were then screened for known homologs with off-target functions (e.g., butyrate-active Mcr homologs, ammonia monooxygenases). Reference sequences were labeled as either true or false positives and then searched against a local copy of the UniRef50 database (release 2019_04) using DIAMOND BLASTp v0.9.24 (49). Any sequences within the Uniref50 database matching either a true or false positive were removed. The labeled true- and false-positive reference sequences were then included in this modified database. The length-curated sequences from IMG/M were searched against the modified database using DIAMOND BLASTp. Sequences for which the top hit was not one of the labeled true positives were removed from further analyses. The remaining sequences from IMG/M were then imported into Geneious and aligned to the reference sequences using MUSCLE, and maximum likelihood trees were inferred with FastTree from the alignments. From these trees, sequences which did not cluster with the reference sequences, were excessively divergent, or were on extremely long branches, were removed.

### Occupancy table construction.

A table containing a row for each metagenome was generated, with columns for each of the six proteins of interest. Each metagenome for which at least one sequence of a given protein remained after curation were marked with a “1” in the appropriate column; otherwise, they were marked with “0.” Here, “1” represents a detection and “0” represents a nondetection, regardless of the total number of detections for a given protein within a given metagenome. These were collectively referred to as the detection histories for each site (Table S2). This table was imported into R v. 3.5.3 along with a table containing metadata for each metagenome, including geographic coordinates (longitude and latitude), ecosystem type (coded as either host-associated, environmental, or engineered), and the date that the samples were uploaded to IMG/M, converted into days since 1 January 2006. These variables were chosen for their completeness on IMG/M. The data were aggregated into three different data sets. The first data set had no aggregation, with each metagenome treated as a separate sample/site. The second data set aggregated metagenomes with identical geographic coordinates into a single site, where any metagenome encoding a gene of interest was sufficient to code that gene as a 1/presence for the given aggregated site. The third data set aggregated metagenomes with identical geographic coordinates and ecosystem types into a single site, under the assumption that a shift from environmental/engineered/host-associated implied a physical separation of samples despite shared geocoordinates (e.g., a sample from a cow rumen and from soil in the same pasture are not likely to be directly impacting one another, compared to samples from different depths in a soil core). This aggregation was achieved using the dplyr v. 0.8.0.1 package (50). Finally, sites with missing metadata were removed to allow comparison of sites.

10.1128/mSystems.00790-21.8 TABLE S2 Occupancy table and metadata for all metagenomes screened from the JGI IMG database (Metadata were curated to near-complete entries, including metagenome ID, ecosystem, longitude, latitude, add date, assembly method, sequencing method, sample date, and the converted numeric add date and converted numeric sample date). Download Table S2, CSV file, 1.2 MB. Copyright © 2021 Hilts et al. 2021 Hilts et al. https://creativecommons.org/licenses/by/4.0/ This content is distributed under the terms of the Creative Commons Attribution 4.0 International license.

### Single-species occupancy modeling.

Single-species occupancy models were used as a preliminary analysis. The species were defined as the functions of interest (methanogenesis and methanotrophy). Surveys (i.e., the repeated sampling events) were defined as the individual genes encoding the enzymes of interest. The R package unmarked v. 0.12-3 (51) was used for occupancy modeling. There were six model sets in total, with a model for each function and each of the aggregated data sets described above. To run the models, data were loaded into unmarkedOccuFrame objects, which combined the detection history data (i.e., the presence/absence table, Table S2) with the site-level metadata. The function occu was used with default parameters, other than the engine parameter, which was set to C. For each set of metagenomes (aggregated or not), model sets were developed using different covariates. The impact of covariates within each model set was compared using the Akaike information criterion (AIC) (52). Where applicable, the occupancy data were plotted against different covariates with 95% confidence intervals.

### Multispecies occupancy modeling.

Multispecies occupancy models were fit according to the Rota et al. (13) model using the function occuMulti in the unmarked package for R in a manner similar to the single-species modeling. Data for both metabolic functions were combined into an unmarkedOccuFrameMulti object. For each data set, models with different parameterizations (Table 1) were run and compared using AIC.

### Data availability.

All sampled metagenomes are publicly available on the Joint Genome Institute’s Integrated Microbial Genomes database. Accession numbers for reference genomes used for positive- and negative-control sequences are listed in Table S1. Accession numbers and accompanying metadata for the metagenomes used are available in Table S2. Supplemental Files 1 to 6 are available on the Open Science Framework (https://doi.org/10.17605/OSF.IO/T97SA). These include an R script which computes and outputs the model statistics (Supplemental File 1). The input table is provided as Supplemental File 2. In addition to the input file, a directory can be specified as an optional parameter, which will save tables of predicted occupancy and error values. These tables were used to generate the figures in this article, and example outputs are included as Supplementary Files 3 to 6.
