# Supplementary figures and images for: Adapting Macroecology to Microbiology: Using Occupancy Modeling To Assess Functional Profiles across Metagenomes
Source: mSystems. 2021 Dec 7;6(6):e00790-21. doi: 10.1128/mSystems.00790-21 (PMC8651082; doi:10.1128/mSystems.00790-21)

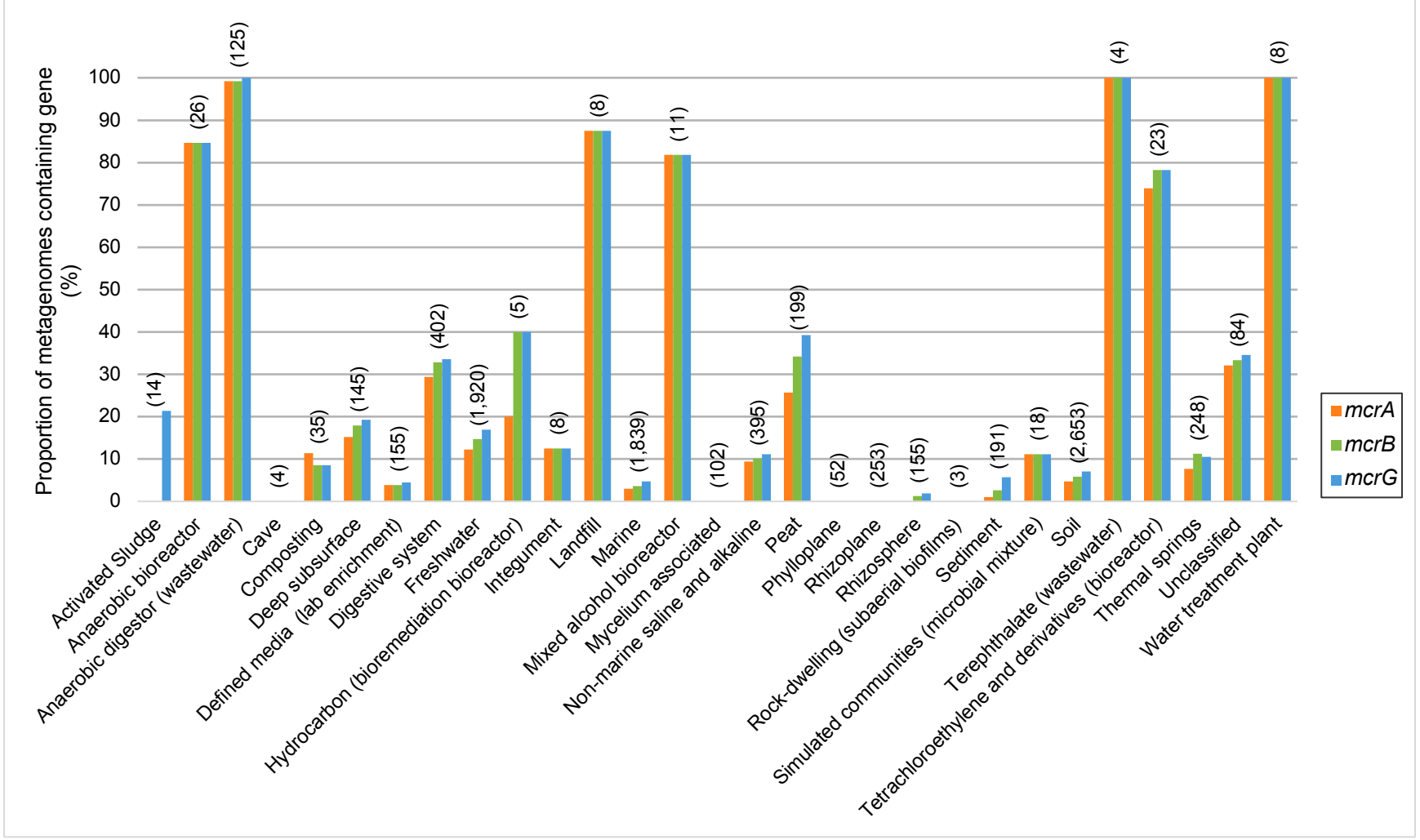

Supplement: FIG S1 [file msystems.00790-21-sf001.pdf]

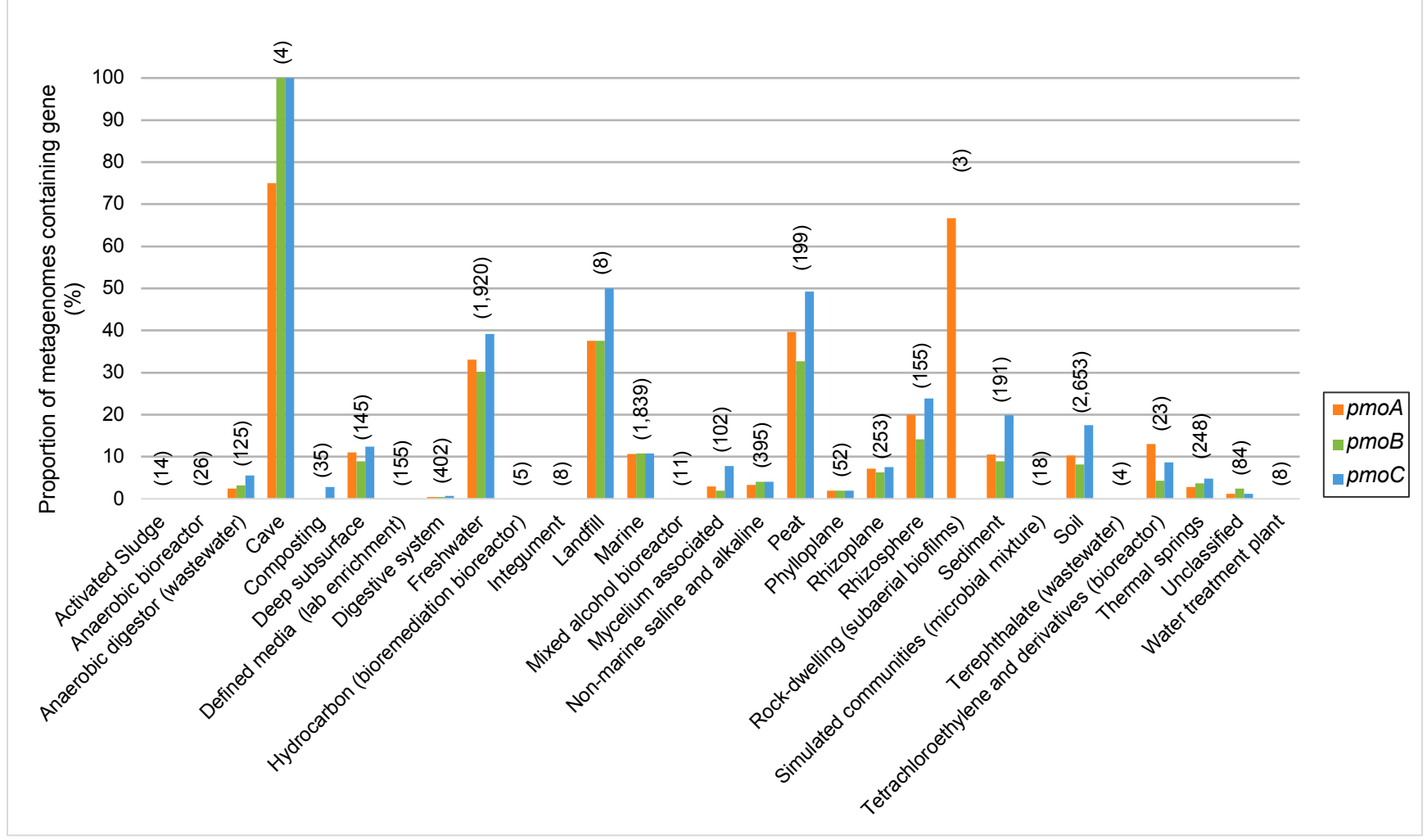

Supplement: FIG S2 [file msystems.00790-21-sf002.pdf]

A: Unaggregated

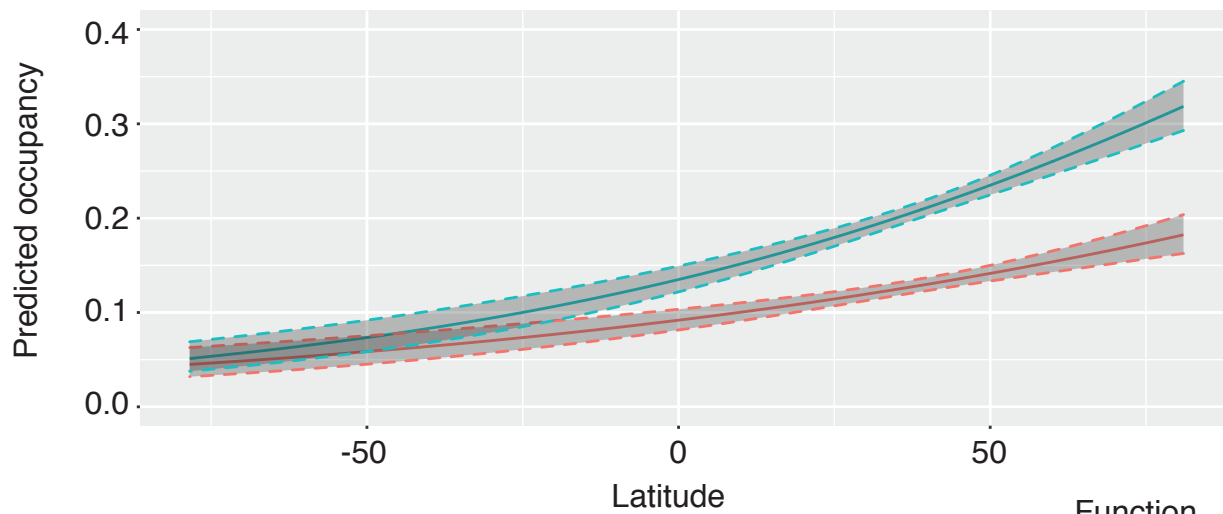

B: Aggregated by geocoordinate

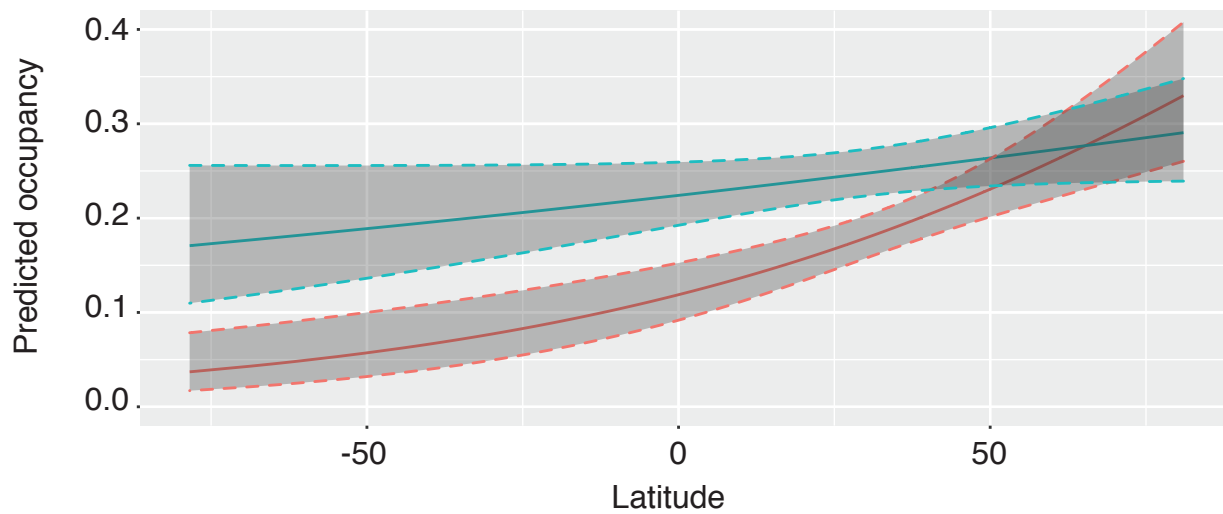

C: Aggregated by geocoordinate and ecosystem

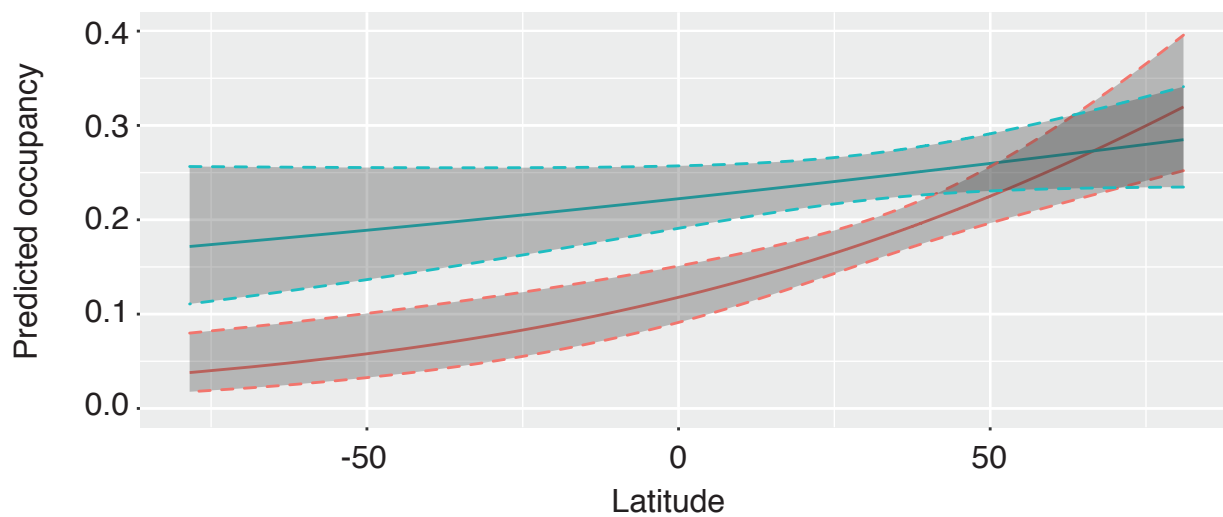

Supplement: FIG S3 [file msystems.00790-21-sf003.pdf]

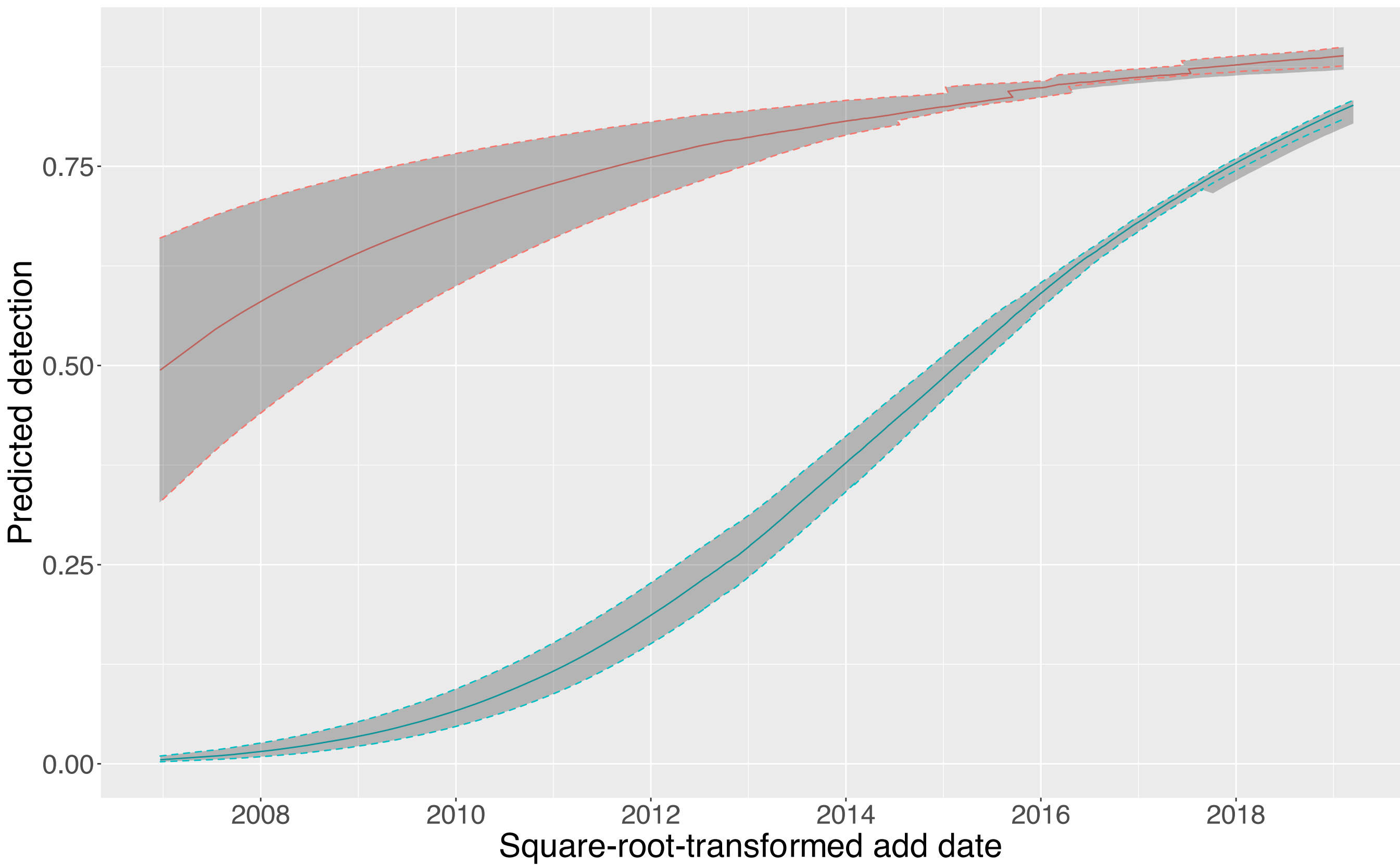

Supplement: FIG S4 [file msystems.00790-21-sf004.pdf]

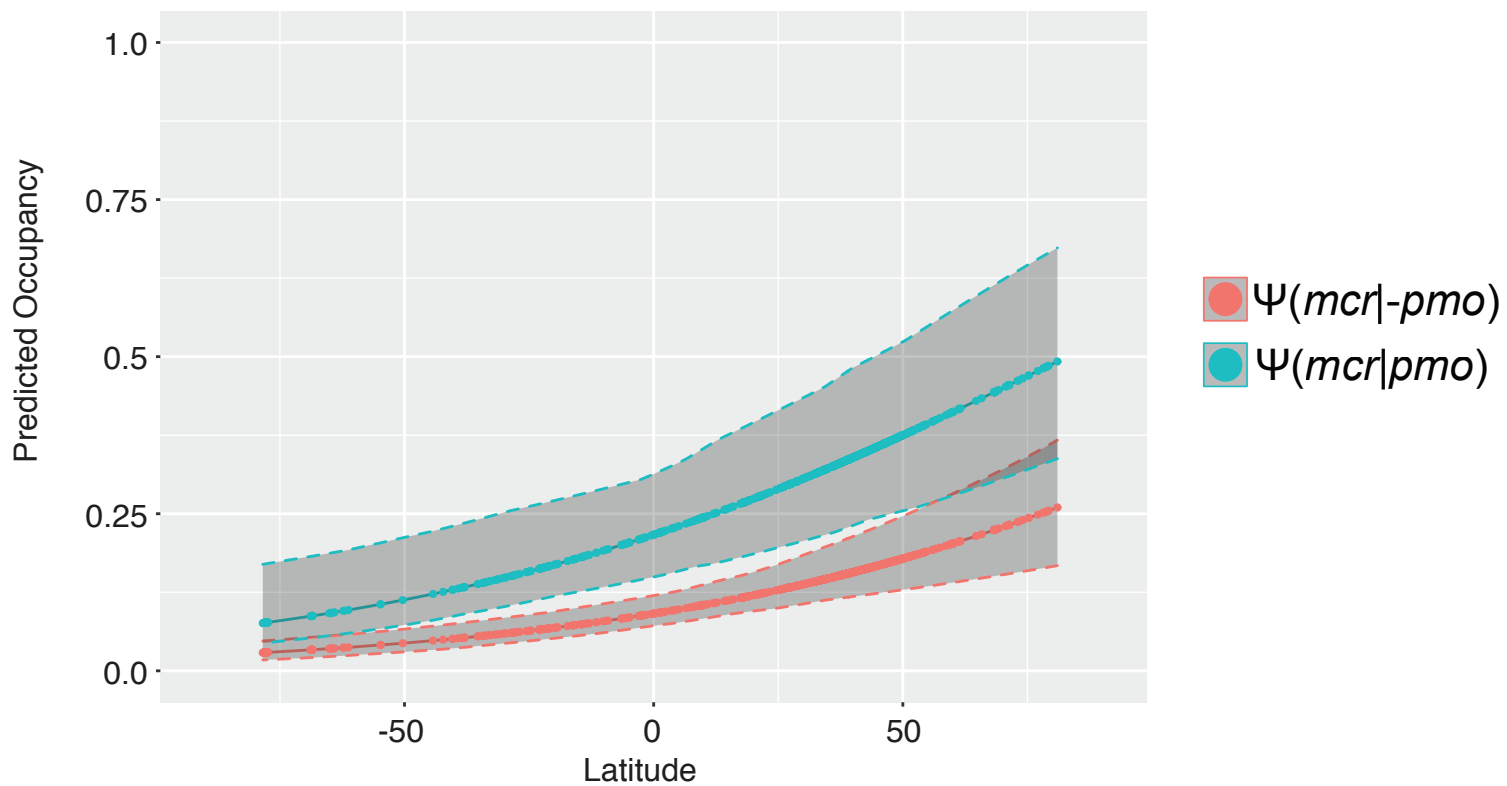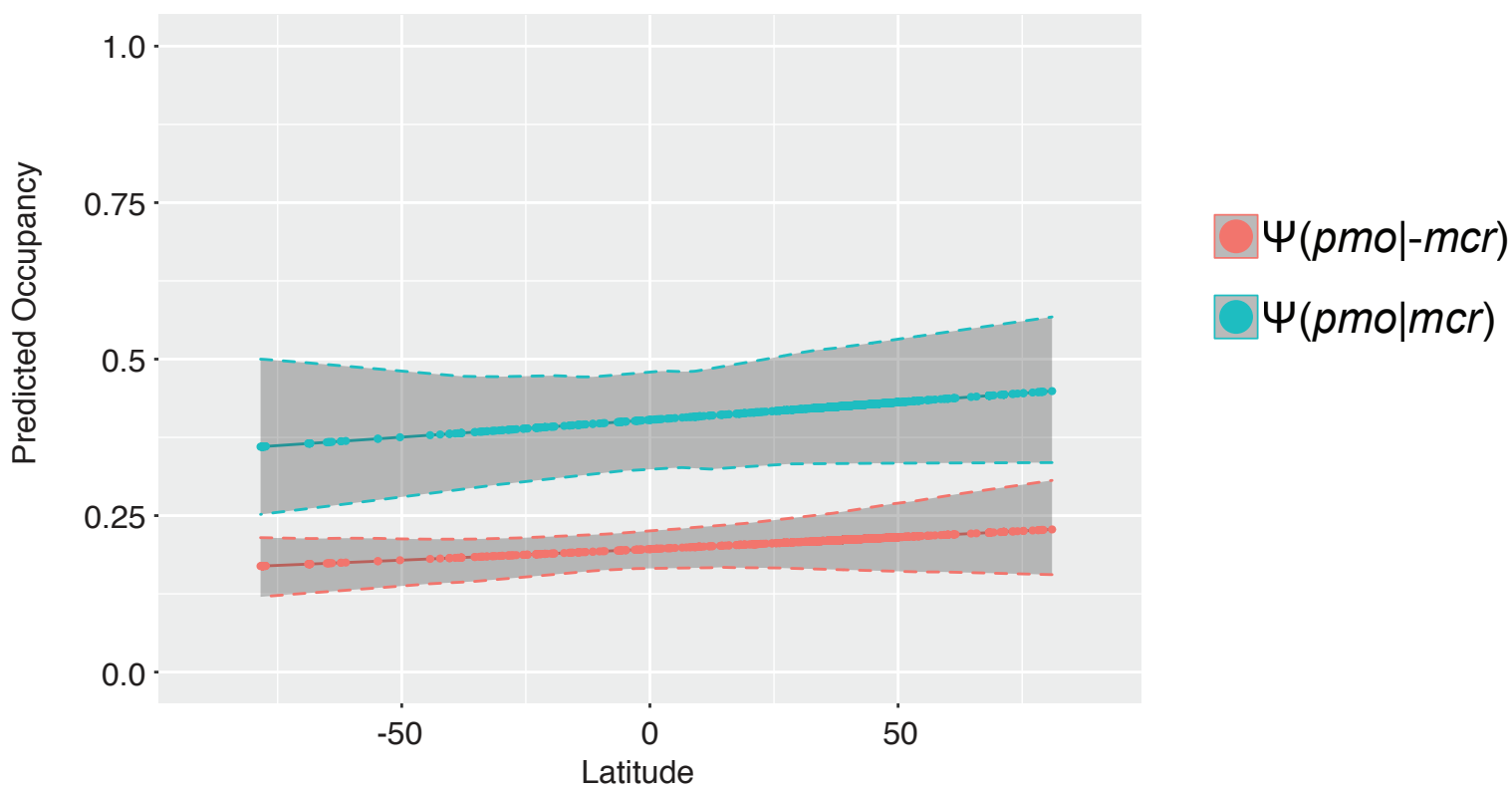

Supplement: FIG S6 [file msystems.00790-21-sf006.pdf]
