# Supplementary material for: Adapting Macroecology to Microbiology: Using Occupancy Modeling To Assess Functional Profiles across Metagenomes
Source: mSystems. 2021 Dec 7;6(6):e00790-21. doi: 10.1128/mSystems.00790-21 (PMC8651082; doi:10.1128/mSystems.00790-21)
Supplement: FIG S5 [file msystems.00790-21-sf005.pdf]

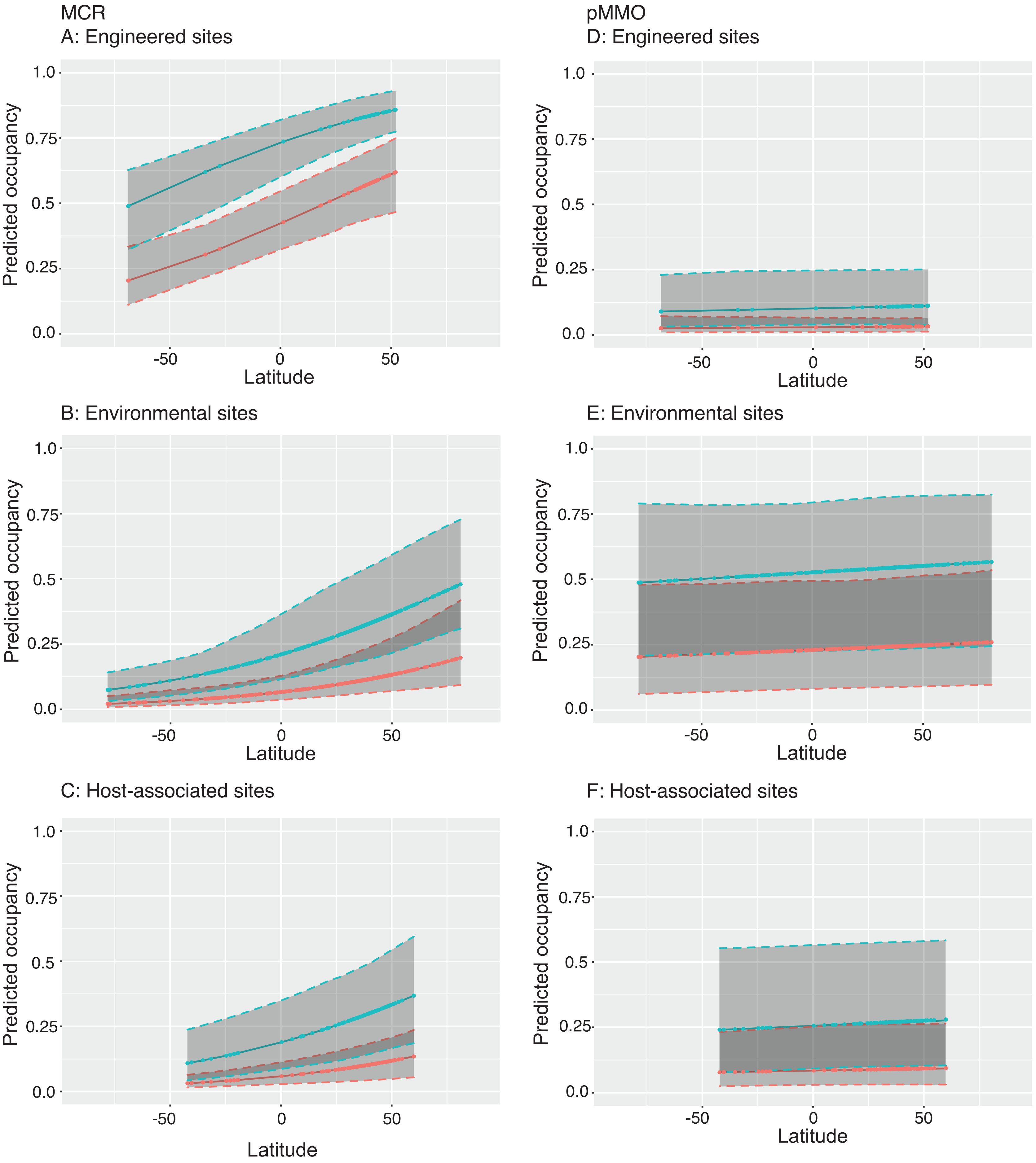
MCR
A: Engineered sites
B: Environmental sites
C: Host-associated sites
pMMO
D: Engineered sites
E: Environmental sites
F: Host-associated sites
Predicted occupancy
Latitude
1.0
0.75
0.5
0.25
0.0
-50
0
50
